# Identifying Risk Factors That Distinguish Symptomatic Severe Acute Respiratory Syndrome Coronavirus 2 Infection From Common Upper Respiratory Infections in Children

**DOI:** 10.7759/cureus.13266

**Published:** 2021-02-10

**Authors:** Jack G Schneider, Ryan F Relich, Dibyadyuti Datta, Caitlin Bond, Michael Goings, Dylan Hall, Guang-Sheng Lei, Jennifer Kedra, Chandy C John

**Affiliations:** 1 Department of Infectious Diseases, Indiana University School of Medicine, Indianapolis, USA; 2 Department of Pathology and Laboratory Medicine, Indiana University School of Medicine, Indianapolis, USA; 3 Department of Pediatrics, Indiana University School of Medicine, Indianapolis, USA; 4 Department of Internal Medicine and Pediatrics, Indiana University School of Medicine, Indianapolis, USA; 5 Department of Pathology and Laboratory Medicine, Indiana University Health, Indianapolis, USA

**Keywords:** covid-19, sars-cov-2, pediatrics, respiratory viral, co-infection

## Abstract

Background

Demographic and clinical risk factors for severe acute respiratory syndrome coronavirus 2 (SARS-CoV-2) infection in children presenting with respiratory viral symptoms are not well defined. An understanding of risk factors for SARS-CoV-2 infection can help prioritize testing.

Methodology

We evaluated potential demographic and clinical factors in children who had respiratory viral symptoms and were tested by polymerase chain reaction (PCR) for SARS-CoV-2 and other respiratory viral infections.

Results

Among the 263 symptomatic children tested for routine seasonal respiratory viruses by PCR, 18 (6.8%) tested positive for SARS-CoV-2. Overall, 22.2% of SARS-CoV-2-infected children and 37.1% of SARS-CoV-2-uninfected children had infection with one or more non-SARS-CoV-2 pathogens (p = 0.31). Higher proportions of children with compared to without SARS-CoV-2 infection were male (77.8 vs. 51.8%, p = 0.05), Hispanic (44.4% vs. 9.8%, p < 0.001), or had the symptoms of fatigue (22.2% vs. 2.5%, p = 0.003) or anosmia/ageusia (11.1% vs. 0%, p = 0.004). History of hypoxic-ischemic encephalopathy (HIE) and obesity were more common in children with versus without SARS-CoV-2 infection (11.1% vs. 1.2%, p = 0.04, and 11.1% vs. 0%, p = 0.004, respectively). In a multivariate analysis, Hispanic ethnicity, symptoms of fatigue or anosmia/ageusia, and presence of obesity (as noted on physical examination) or HIE were independently associated with SARS-CoV-2 infection. Numbers in each category were small, and these preliminary associations require confirmation in future studies.

Conclusions

In this area of the United States, infection with other viruses did not rule out infection with SARS-CoV-2. Additionally, children with respiratory viral symptoms who were of Hispanic ethnicity, had symptoms of weakness/fatigue, or had obesity or HIE were at an increased risk for SARS-CoV-2 infection. Future studies should assess if these factors are associated with risk in populations in other areas of the United States.

## Introduction

The sudden emergence and rapid global spread of severe acute respiratory syndrome coronavirus 2 (SARS-CoV-2), the etiologic agent of coronavirus disease 2019 (COVID-19), led to a global pandemic. By May 2020, the United States emerged as the country with the highest burden of COVID-19. There is limited data on demographic and clinical risk factors for SARS-CoV-2 infection or risk of viral co-infections in children in the United States. The few studies done on co-infection or symptoms in children have assessed cohorts of children with SARS-CoV-2 infection [1-5] and have not evaluated risk factors or prevalence of co-infections in children with respiratory viral symptoms with versus without SARS-CoV-2 infection.

We conducted this study to determine demographic and clinical risk factors for SARS-CoV-2 infection in children with respiratory viral symptoms who were evaluated for SARS-CoV-2 and other respiratory viral infections by nasopharyngeal swab polymerase chain reaction (PCR) testing. The study represents a real-world assessment of the prevalence of SARS-CoV-2, and risk factors for SARS-CoV-2, in children with respiratory viral symptoms significant enough to lead an Emergency Department (ED) physician to order respiratory viral panel (RVP) PCR testing. During the time period of this study, there was no mandated or recommended SARS-CoV-2 testing in children, and almost all children with respiratory symptoms significant enough to warrant RVP testing were also tested for SARS-CoV-2 infection.

## Materials and methods

Study design, population, and setting

Between March 25th and May 17th, 2020, we conducted a retrospective evaluation of risk factors for SARS-CoV-2 infection in children in whom RVP PCR testing was ordered by a physician at either Riley Hospital for Children in Indianapolis or one of the other 14 satellite hospitals in the Indiana University Health network throughout the state. The indication for RVP testing is the presence of respiratory viral symptoms, such as fever, cough, or rhinorrhea, but there was no definitive checklist of symptoms as the testing was done at the discretion of the ED physician. Additionally, there was no mandated or recommended SARS-CoV-2 testing in children during the time period of the study. SARS-CoV-2 testing was done only if suggested by specific symptoms. Collected nasopharyngeal (NP) swabs were submitted to the Indiana University Health Division of Clinical Microbiology in Indianapolis, Indiana, for a routine RVP. Each child was included as a unique participant in the analysis. Among participants with additional tests reported, data were used from the test with positive SARS-CoV-2 result or, if SARS-CoV-2 negative and RVP positive, the positive RVP test for analysis from the encounter when the test was performed. Prevalence of SARS-CoV-2 and seasonal respiratory virus co-infections were calculated, and medical chart reviews were performed to assess whether routine laboratory parameters, specific symptoms, or sex/age correlated with an increased likelihood of SARS-CoV-2 PCR being positive. Additionally, prevalence of SARS-CoV-2 positivity by PCR over the eight-week study period among children tested by RVP were compared to all children not tested by RVP (the same time frame and health facilities) but specifically tested for SARS-CoV-2 by PCR.

Study samples and respiratory pathogen testing

NP swab specimens were collected on flocked nylon swabs (Copan or Puritan) and submitted in viral transport medium (VTM; MicroTest M6 Multi-Microbe Media, Remel; Universal Transport Medium, Copan; in-house transport medium, CDC-endorsed). If standard-of-care (SOC) testing could not be performed immediately following receipt by the laboratory, specimens were stored at 4°C. All specimens were tested within eight hours of receipt by the laboratory. For seasonal respiratory virus detection, all specimens were tested by the FilmArray Respiratory Panel 2 (RP2; BioFire Diagnostics, Salt Lake City, UT, USA). For SOC SARS-CoV-2 testing, either the NxTAG CoV Extended Panel (Luminex, Austin, TX, USA) or the cobas SARS-CoV-2 Test (Roche, Basel, Switzerland) was used. Remnant VTM was stored at -80°C. For detection of SARS-CoV-2 in the 17 specimens that did not have orders for SOC SARS-CoV-2 testing (but tested by RVP), aliquots of thawed remnant VTM were manually extracted using the Quick-DNA/RNA Viral Kit (Zymo Research) and extracts were analyzed by the real-time RT-PCR protocol developed by Corman et al. for detection of the SARS-CoV-2 E and RdRp genes using the Rotor-Gene Q (QIAGEN, Hilden, Germany) system [6].

Clinical markers, symptoms, and co-morbidities associated with respiratory viral infections

Electronic medical records were reviewed to determine the clinical markers (white blood cell [WBC] count, absolute lymphocyte and neutrophil counts, platelets, alanine aminotransferase, aspartate aminotransferase, total bilirubin, and procalcitonin) and reported symptoms (e.g., fever, cough, shortness of breath, etc.) commonly associated with acute respiratory infections in symptomatic pediatric patients. We also evaluated the prevalence of preexisting co-morbidities to determine the risk factors associated with the differential risk of testing positive from respiratory virus infections.

Statistical analysis

Differences in proportions of specimens positive for non-SARS-CoV-2 respiratory pathogens according to the presence of SARS-CoV-2 infection were compared using Fisher’s exact test. Demographic, clinical characteristics, and laboratory values of children who tested positive for SARS-CoV-2 versus those who were negative were compared using Fisher’s exact test for categorical variables and Wilcoxon rank-sum test for continuous variables. The Wilcoxon rank-sum test was used because the continuous variables were not normally distributed. Penalized maximum likelihood estimation, as proposed by Firth [7], was used to estimate independent predictors of risk of SARS-CoV-2 infection in a multivariate logistic regression model. This method was used because it allows estimation of odds ratios (ORs) in cells with few or no subjects. A final penalized maximum likelihood estimation multivariate logistic regression model was fit using stepwise backward selection, starting with all significant variables from univariate models. All analysis was done in Stata 14.2 (Stata Corporation, College Station, TX, USA), and penalized maximum likelihood estimation was performed using the Stata program firthlogit by Joseph Coveney [8].

Ethical review

Ethical review and approval were given by the Indiana University Institutional Review Board (IRB). The IRB granted expedited review to perform chart evaluation for the variables studied.

## Results

Study screening and inclusion

**Figure 1:**
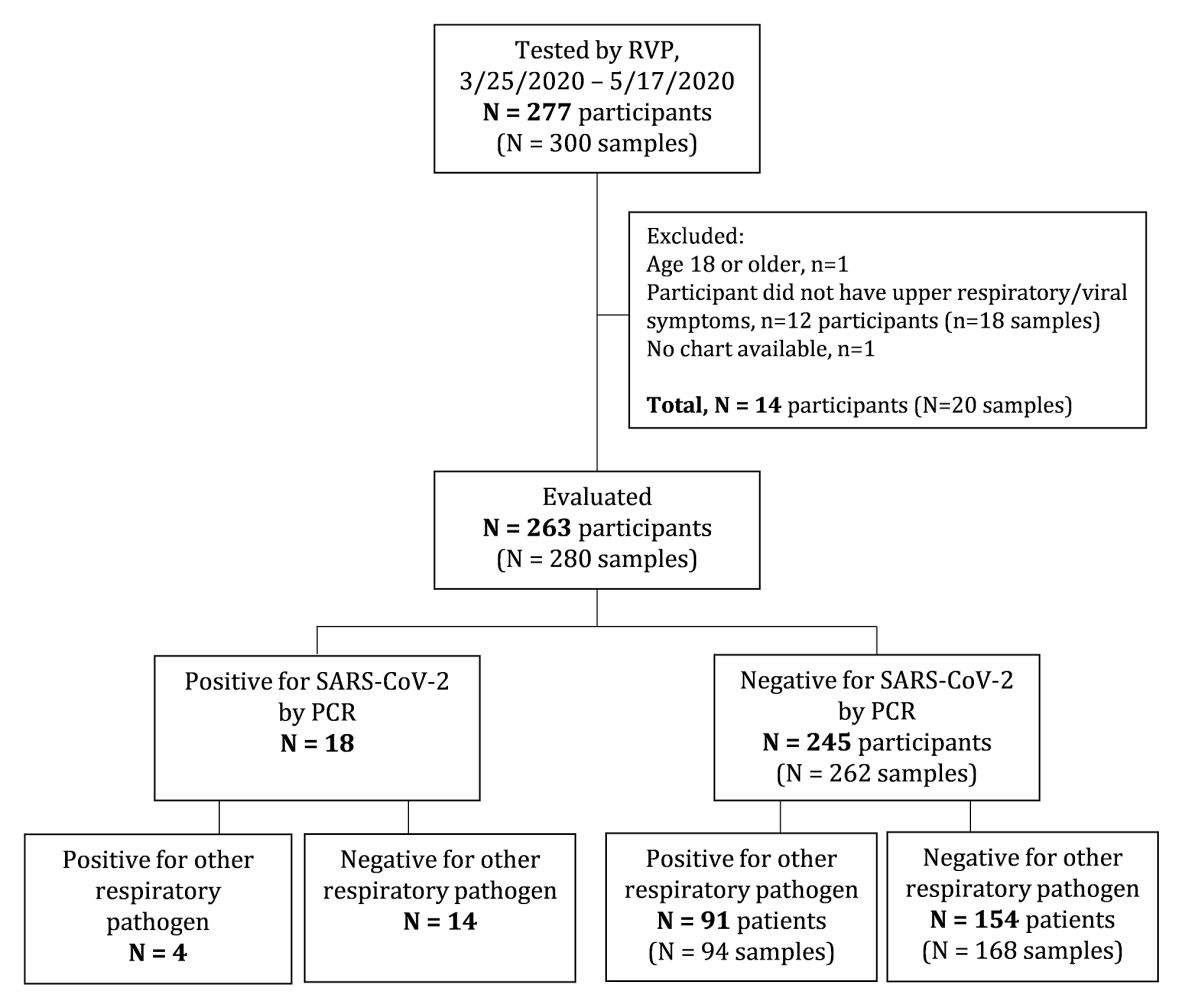
Study participant flow diagram.

Indiana, including Indianapolis, was endemic for SARS-CoV-2 infection during the time period of the study, with a total of 27,642 cases of COVID-19 reported to the Indiana State Department of Health over the study period, including 8,094 cases in Marion County/Indianapolis [9]. We screened 277 symptomatic children for study inclusion, of whom 263 participants <18 years of age were deemed eligible for inclusion, and NP swabs were collected for testing. Details about exclusion criteria and study enrollment numbers are included in Figure 1. Of the 263 children in the study, 18 (6.8%) were positive for SARS-CoV-2, and 16 of these 18 children (88.9%) required inpatient care, including four of 16 (25.0%) who required intensive care unit (ICU) care. A total of 91 (34.6%) children were positive for other respiratory pathogens, and 154 (58.6%) were negative for all respiratory pathogens. Among the 245 children who were negative for SARS-CoV-2 infection, 211 (86.1%) were admitted to the hospital, and of those, 40 (19.0%) required ICU-level care. Thus, the rates of admission and ICU care were similar in children with respiratory symptoms with versus without SARS-CoV-2 infection. Among the 91 participants who tested positive for routine seasonal respiratory viruses, three received repeat NP swab testing for new or persistent upper respiratory/viral symptoms, which were negative for SARS-CoV-2 as well as other respiratory viruses. Among the 154 participants with initial negative respiratory virus test results, 14 had repeat NP swabs collected for new or persistent respiratory viral symptoms and were also all negative on follow-up testing. Three participants in the SARS-CoV-2-positive group were negative on initial NP testing but positive on follow-up testing during the same inpatient illness (interval range between repeated tests were three, seven, and 14 days). For these participants, data from the follow-up testing time-point is reported in this study.

Higher proportion of SARS-CoV-2-infected children were Hispanic/Latinx

**Figure 2:**
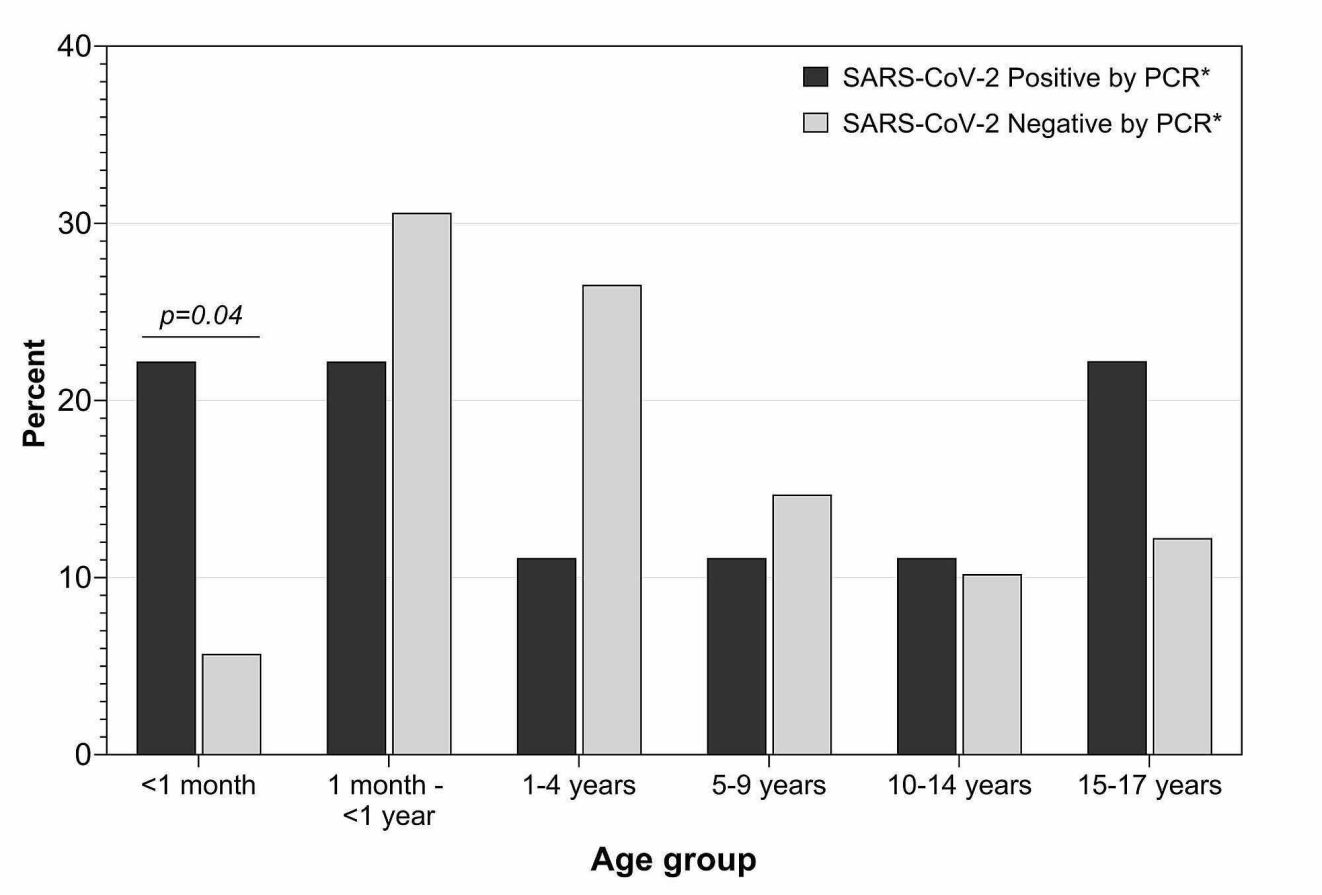
Age distribution of children tested for respiratory viral pathogens according to SARS-CoV-2 infection status. SARS-CoV-2, severe acute respiratory syndrome coronavirus 2; PCR, polymerase chain reaction P-value from Fisher’s exact test to compare age distribution according to SARS-CoV-2 infection among children under one year of age (n = 97). Overall, no significant difference across all age groups *PCR platforms used: NxTAG CoV Extended Panel; Cobas SARS-CoV-2 Test; or Quick-DNA/RNA Viral Kit

There were no significant differences in median age (Table 1) between children tested by RVP who had SARS-CoV-2 infection compared to those without SARS-CoV-2 infection. Among those less than one year of age, 50% (four of eight) with SARS-CoV-2 infection were under one month of age, whereas only 15.7% (14 of 89) of those testing negative for SARS-COV-2 were under one month of age (p = 0.04) (Figure 2). There was slightly higher proportion of males than females among SARS-CoV-2-infected versus uninfected children (77.8 vs. 51.8%, p = 0.05). A substantially higher proportion of SARS-CoV-2-infected children versus children not infected with SARS-CoV-2 were Hispanic/Latinx (44.4 vs. 9.8%, p < 0.001, Table 1).

**Table 1:** Patient characteristics according to SARS-CoV-2 testing result by PCR. SARS-CoV-2, severe acute respiratory syndrome coronavirus 2; PCR, polymerase chain reaction; No., number; IQR, interquartile range; y, year ^a^ Data represent number and percentage of children unless otherwise specified ^b^ Fisher’s exact test for categorical variable comparisons and Wilcoxon rank-sum test for continuous variable (age) were used ^c^ Four are White with unknown ethnicity ^d^ Bradycardia Abbreviations: PCR, polymerase chain reaction; IQR, interquartile range

Characteristic	SARS-CoV-2 testing by PCR, No. (%)^a^		
	Positive	Negative	P-Value^b^
No. of participants	18	245	
No. of samples	18	262	
Age, median (IQR), y	2.78 (0.10, 14.02)	2.21 (0.62, 8.79)	0.72
Sex, female	4 (22.22)	118 (48.16)	0.05
Race/Ethnicity			
White, non-Hispanic	2 (11.11)	139 (56.73)	<0.001
Black, non-Hispanic	6 (33.33)	57 (23.27)	0.39
Hispanic or Latinx	8 (44.44)	24 (9.80)	<0.001
Asian, Pacific Islander	1 (5.56)	2 (0.82)	0.19
Other race	1 (5.56)	1 (0.41)	0.13
Unknown race or ethnicity^c^	0	22 (8.98)	0.38
Symptoms			
Fever	10 (55.56)	140 (57.14)	0.99
Cough	7 (38.89)	99 (40.41)	0.99
Shortness of breath	11 (61.11)	100 (40.82)	0.14
Nasal congestion/Rhinorrhea	5 (27.78)	56 (22.86)	0.58
Sore throat	1 (5.56)	10 (4.08)	0.55
Nausea/Vomiting	5 (27.78)	54 (22.04)	0.56
Diarrhea	0	16 (6.53)	0.61
Fatigue/Malaise/Weakness	4 (22.22)	6 (2.45)	0.003
Myalgia/Arthralgia	1 (5.56)	7 (2.86)	0.44
Rash/Skin lesions	0	17 (6.94)	0.62
Anosmia/Ageusia	2 (11.11)	0	0.004
Subjective fevers	1 (5.56)	9 (3.67)	0.51
Seizures	3 (16.67)	16 (6.53)	0.13
Other	1 (5.56)^d^	11 (4.49)	0.58

Children with SARS-CoV-2 infection reported more fatigue and anosmia/ageusia

The most common symptoms, fever, cough, and shortness of breath, were reported in >40% of participants and did not differ between SARS-CoV-2-infected children and children not infected with SARS-CoV-2 (Table 1). Fatigue/malaise/weakness and anosmia/ageusia were more common in SARS-CoV-2-infected children and children not infected with SARS-CoV-2 (22.2 vs. 2.5%, p = 0.003 and 11.1 vs. 0%, p = 0.004, respectively, Table 1).

Co-infection with other respiratory viruses did not differ significantly between SARS-CoV-2-infected and SARS-CoV-2-uninfected children

Among the 18 participants positive for SARS-CoV-2 infection, four (22.2%) were co-infected with other respiratory viral pathogens (Appendix, Table 3) compared to 91/245 (37.1%) SARS-CoV-2-uninfected children (p = 0.31). There were no major differences in symptoms or clinical features between four children who were positive for SARS-CoV-2 and other respiratory viral pathogens versus the 14 children with SARS-CoV-2 and without any co-infection (Appendix, Table 4).

SARS-CoV-2 prevalence varied over time, but with no trend toward an overall decrease or increase

**Figure 3:**
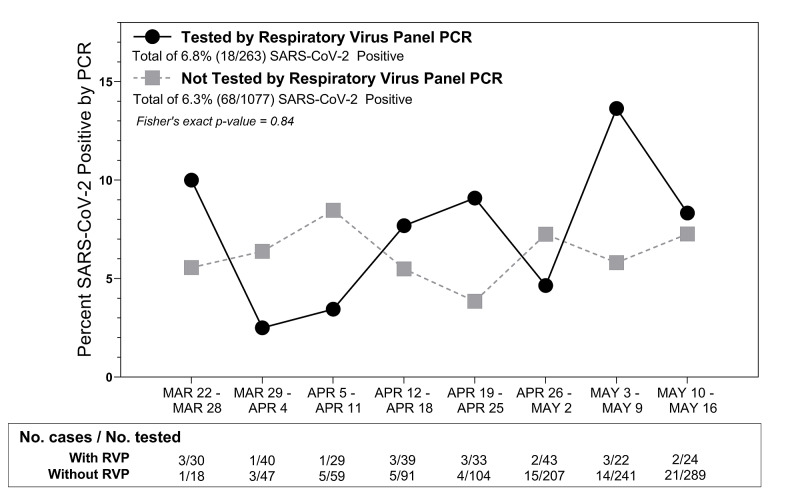
Prevalence of SARS-CoV-2 by PCR over the eight-week study period among children tested by RVP (n = 263) compared to children not tested by RVP (n = 1077). SARS-CoV-2, severe acute respiratory syndrome coronavirus 2; PCR, polymerase chain reaction; RVP, respiratory viral panel The study period began in the middle of the week on March 25th. The last day of the study was Sunday, May 17th (not shown in figure), with no positive SARS-CoV-2 by PCR among children with RVP (n = 3) or without RVP (n = 21)

The prevalence of SARS-CoV-2 infections over time in this cohort of children who were tested for other respiratory viral pathogens (6.8%) was comparable to the prevalence over time for children who were not tested with RVP test (6.3%) but tested specifically for SARS-CoV-2 by PCR (Figure 3). In the eight weeks during which we conducted this study, there was weekly variability in the SARS-CoV-2 prevalence, but no trend toward an increase or decrease over time (Figure 3).

Clinical laboratory values did not differ significantly between SARS-CoV-2-infected and uninfected children

SARS-CoV-2-infected children had slightly lower WBC count than those who were not SARS-CoV-2 infected (Table 2), but the median WBC count was within normal limits in both groups. No other laboratory values differed significantly.

**Table 2:** Laboratory values in children with versus without SARS-CoV-2 infection. SARS-CoV-2, severe acute respiratory syndrome coronavirus 2; PCR, polymerase chain reaction; IQR, interquartile range; WBC, white blood cells; CRP, C-reactive protein; ALT, alanine aminotransferase; AST, aspartate aminotransferase ^a^ Wilcoxon rank-sum test; p-values reported

	SARS-CoV-2 PCR status, Median (IQR) (N)		
	Positive	Negative	p-value^a^
WBC (k/cumm)	7.7 (6.7, 8.8) (9)	11.3 (7.1, 15.4) (178)	0.05
Absolute lymphocyte count (k/cumm)	3.4 (1.7, 4.2) (9)	2.5 (1.2, 4.2) (168)	0.31
Absolute neutrophil count (k/cumm)	3.1 (2.7, 4.9) (9)	5.8 (3.2, 10.6) (169)	0.07
Platelets (k/cumm)	267 (191, 350) (9)	298 (200, 391) (177)	0.63
CRP (mg/dL)	2.6 (1.1, 9.5) (13)	3.6 (1.2, 14.2) (49)	0.59
ALT (Units/L)	15 (15, 27) (9)	20 (13, 30) (118)	0.74
AST (Units/L)	34 (33, 35) (9)	32 (24, 48) (119)	0.82
Total bilirubin (mg/dL)	0.30 (0.25, 0.40) (8)	0.45 (0.30, 0.80) (118)	0.07
Procalcitonin (ng/mL)	0.06 (1)	0.12 (0.09, 0.17) (5)	0.14

Comorbidities were frequent in children with respiratory viral infections, and obesity and hypoxic-ischemic encephalopathy were more frequent in SARS-CoV-2-infected children

Co-morbidities were common in children with respiratory viral symptoms who were tested by RVP. A higher proportion of children with SARS-CoV-2 versus without SARS-CoV-2 infection had hypoxic-ischemic encephalopathy (HIE) or obesity (11.1 vs. 1.2%, p = 0.04, and 11.1 vs. 0%, p = 0.004; Appendix, Table 5).

Hispanic/Latinx ethnicity, obesity, history of hypoxic-ischemic encephalopathy, and symptoms of fatigue/malaise/weakness and anosmia/ageusia were independently associated with SARS-CoV-2 infection

**Figure 4:**
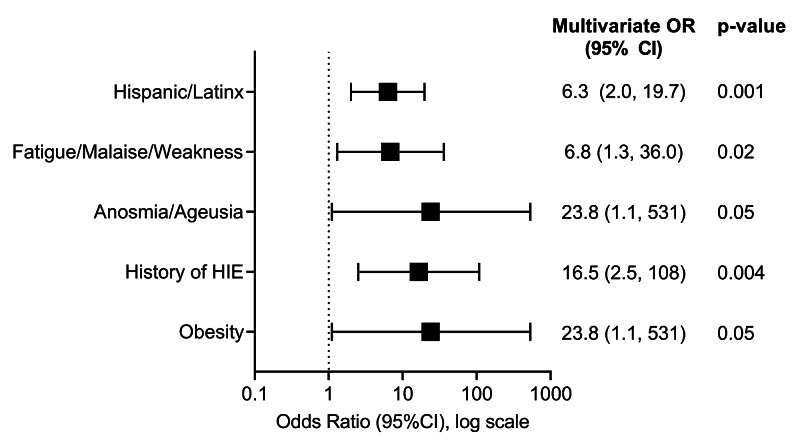
Predictors of positive SARS-CoV-2 result by PCR using penalized maximum likelihood multivariate logistic regression. SARS-CoV-2, severe acute respiratory syndrome coronavirus 2; PCR, polymerase chain reaction; HIE, hypoxic-ischemic encephalopathy; OR, odds ratio; CI, confidence interval

In a multivariate analysis including demographic and clinical factors significantly associated with SARS-CoV-2 infection in univariate analysis, Hispanic/Latinx ethnicity (OR = 6.3, 95% confidence interval [CI]: 2.0-19.7), obesity (OR = 23.8, 95% CI: 1.1-531), history of HIE (OR = 16.5, 95% CI: 2.5-108), and presence of fatigue/malaise/weakness (OR = 6.8, 95% CI: 1.3-36) or anosmia/ageusia (OR = 23.8, 95% CI: 1.1-531) were independently associated with increased risk of SARS-CoV-2 infection (Figure 4). Prevalence of obesity, history of HIE, anosmia/ageusia, or fatigue/malaise/weakness did not differ significantly between Hispanic and non-Hispanic children (data not shown).

## Discussion

Studies in children with SARS-CoV-2 have typically assessed cohorts with SARS-CoV-2 infection, without a comparison group of children with respiratory viral symptoms without SARS-CoV-2 infection. Without the comparison group, it is not possible to assess differences in the risk of SARS-CoV-2 infection with specific demographic or clinical factors in children with respiratory viral symptoms. In the current era of persistently limited testing capacity, and with a current respiratory viral season in the United States, identification of factors that predict an increased likelihood of SARS-CoV-2 infection could help with prioritization of testing in children with respiratory viral symptoms. Given widespread testing is key to contact tracing and control, knowing predictable factors for SARS-CoV-2 infection would allow for the conservation of resources, especially in settings with low testing capabilities. In the present study, we show that Hispanic/Latinx ethnicity, obesity, history of HIE, and symptoms of fatigue and anosmia/ageusia were independent risk factors for SARS-CoV-2 infection. We also show that presence of other respiratory viral pathogens did not differ in children with versus without SARS-CoV-2 infection. If our findings are confirmed by larger studies in other areas of the United States, they would support prioritization of SARS-CoV-2 testing in children with Hispanic/Latinx ethnicity, obesity, history of HIE, symptoms of fatigue/malaise/weakness, or anosmia/ageusia.

Studies among adults have shown that the presence of other respiratory viral infections does not exclude SARS-CoV-2 infection [10,11]. Our study adds to this literature by showing the same is true in children, as earlier studies conducted in children have evaluated co-infections only in SARS-CoV-2-positive patients [3,5]. The rate of viral co-infections was 22.2%, comparable to the rate in adults in a study by Kim et al. (20.7%) [10]. The most common co-infection in our patients positive for SARS-CoV-2 were rhinovirus/enterovirus (11.1%) and non-SARS-CoV-2 Coronaviridae: NL63 (11.1%), while for non-SARS-CoV-2 patients rhinovirus/enterovirus (24.1%), adenovirus (7.8%), and human metapneumovirus and respiratory syncytial virus (both 4.1%) were most common. While no children in our study had SARS-CoV-2 and influenza co-infection, a study by Stowe et al. found that 6.1% of children with SARS-CoV-2 were co-infected with influenza towards the end of the influenza season in England [5], suggesting that co-infection rates could increase during influenza season in the United States. Further studies that include seasonal variation of respiratory viruses are needed to fully define the spectrum of respiratory viral co-infection.

Studies in adults have shown a higher frequency of SARS-CoV-2 infection in Hispanic and African American adults [12,13]. Some pediatric studies have reported a high frequency of Hispanic ethnicity in children with SARS-CoV-2 infection [1,14], but these studies lacked a comparator group of children with respiratory viral symptoms who did not have SARS-CoV-2 infection to allow analysis of relative frequency of infection. Higher Hispanic or African American representation among children with SARS-CoV-2 infection could simply reflect the predominant ethnicity/race in the area. In the present study, we show that almost half of the SARS-CoV-2-infected children were Hispanic, and that Hispanic ethnicity was associated with a four-fold increased risk of SARS-CoV-2 infection in children with upper respiratory/viral symptoms. Because of the limited time frame of the study, it is possible that the higher risk for SARS-CoV-2 disease in Hispanic children was due to local outbreaks that occurred within these communities, and that over a longer time, this association might not be seen. However, other studies also showing a higher risk of SARS-CoV-2 disease in Hispanic adults [13] and children [1,15] suggest that the association is not due solely to clustering during an outbreak.

As in adults, structural determinants of health and the effects of systemic racism may lead to problems with healthcare access, access to high quality nutrition, access to education and care for chronic health problems, and other factors that may increase the risk of disease with COVID-19 [15]. In addition, the effects of the same factors on the adult caregivers of these children may place them at greater risk for COVID-19, and therefore at greater risk of passing this infection on to their children [16]. Genetic factors could potentially play a role in disease risk, but the Hispanic community is genetically diverse, and to date, genetic risk factors among Hispanic children and adults have not been defined. The study provides further support for addressing root causes of discrimination, racism, and lack of access to adequate nutrition and health care in underrepresented minorities [17,18].

In our study, obesity was noted to be more frequent in children with SARS-CoV-2 infection, which is important given its recent association for higher mortality and disease severity in adults [19] and children [1]. However, as height information was missing on many patients, obesity was based on notation of obesity in the physical examination, rather than by body mass index (BMI) calculation. However, the overall number of patients (n = 2) noted to be obese was small, and examination notation without BMI confirmation is not a rigorous way of assessing BMI; hence, these results should be interpreted with caution. Fatigue/malaise/weakness and anosmia/ageusia were reported more frequently in SARS-CoV-2-infected children. Fatigue/malaise/weakness, not previously described as a more common symptom, may be worth including in systematic symptom assessment of children with upper respiratory/viral symptoms, though its lack of specificity may make assessment difficult. Anosmia and ageusia have been reported in many adults [20-22], but only recently reported in children [2,23,24]. These very specific symptoms should be asked for in all children with upper respiratory/viral symptoms. In this study, they were more frequent, but by self-report, that is, they were not systematically asked. The symptoms might have been even more common if every patient was asked this question. Follow-up studies should also determine how long this persists in children, as in most adults it resolves over time [20].

A history of HIE and its association with SARS-CoV-2-infection has not been well described in children. While a case series involving patients with SARS-CoV-2 infection and Down syndrome included one patient with a history of HIE and seizures, there are no other reports associating HIE as an at-risk category for SARS-CoV-2 infection [25]. It is important to note that HIE may not provide the best insight into true co-morbidity. While it was not grouped with other individuals with neuromuscular or respiratory diseases in the analysis, we chose to separate this condition given its potential risk for SARS-CoV-2 infection. Similar to obesity, this finding will need to be confirmed in further studies with a larger number of patients.

As in previous studies, age, laboratory parameters, and other clinical factors did not differ between SARS-CoV-2-infected and uninfected children in our study [3,26,27]. Lymphopenia has been described in hospitalized children [28] and in adult cases [29], but it was not a prominent clinical feature in our diverse group of patients. It is important to mention that even though the comorbidity percentages between both the SARS-CoV-2-positive and negative groups were similar, patients uninfected with SARS-CoV-2 included more immunosuppressant use and underlying hematology/oncology diagnoses, which could have potentially altered laboratory findings between the two groups. Although all age groups were infected with SARS-CoV-2, our data revealed infants being more susceptible to infection with a bimodal distribution of patients <1 year (44.4%) and those between 15 and 17 years of age (22.2%), similar to recently reported case series in children [4,30].

Study strengths include assessment of prevalence of SARS-CoV-2 infection over time, and assessment of risk factors in all children tested for respiratory viral infections, not just those with documented SARS-CoV-2 infection. This assessment allowed us to determine independent risk factors for SARS-CoV-2 infection in children, as well as the magnitude of association with each factor.

The main limitations of our study were the small sample size of positive SARS-CoV-2 infections, which was notable in children early in the pandemic, and that our comparison groups were not matched. However, several strong associations were found, and the study lays the groundwork for these to be compared in future, larger studies. Our study was also limited by a short study time frame early in the pandemic (does not allow for evaluation of seasonal effects on viral transmission), the single site of evaluation, and the possibility that different health-seeking behaviors in various populations might affect the likelihood of being in the study. Importantly, however, this study did include all children who were tested for respiratory viral infections in this time period, so health-seeking behavior alone would not explain the differences seen in the risk factors for SARS-CoV-2 among those tested.

When taking into account why providers ordered an RVP versus a stand-alone SARS-CoV-2 PCR test, it is important to understand our study was performed during the early stages of the pandemic, prior to the addition of timely testing platforms, and without clear clinical criteria for SARS-CoV-2 diagnosis. RVP and SARS-CoV-2 testing were ordered separately because there was no combined test, and 94.3% (283/300) of children who had an RVP test also had SARS-CoV-2 PCR testing, so almost all children with RVP testing had SARS-CoV-2 testing ordered at the same time. There was no rationale for ordering an RVP without PCR. The small number who did not have SARS-CoV-2 testing ordered (17 children) had the testing done subsequently on a sample stored at -80°C. In this small group, SARS-CoV-2 was likely not ordered because of reagent shortage or testing back-up, rather than for any clinical rationale. Because physicians were routinely sending both tests early in the pandemic on those patients with viral respiratory symptoms, patients specifically with underlying chronic conditions were likely to have similar testing done if they had viral respiratory symptoms. It is also important to note that other satellite ED sites throughout the state may have had different ordering practices and were not staffed with pediatricians.

## Conclusions

The study results demonstrate that the presence of other respiratory viral pathogens does not rule out SARS-CoV-2 co-infection in children and suggest that Hispanic children are at a particularly high risk of SARS-CoV-2 infection. Anosmia and ageusia were more common with SARS-CoV-2 infection in children, as in adults, and should be asked about in all children evaluated for respiratory symptoms in COVID-19-endemic areas. Further studies are required to confirm these preliminary results with study samples being obtained over a longer period of time and in multiple areas given the wide variation in viral epidemiology throughout the year. The data generated from this study can help to guide EDs on evaluation for SARS-CoV-2 infection in children with respiratory viral symptoms and highlight the need for better evaluation of why COVID-19 is so much more frequent in Hispanic children, including evaluation of the contribution of racism to this risk.

## Appendices

**Table 3:** Proportions of specimens positive for non-SARS-CoV-2 respiratory pathogens according to the presence of SARS-CoV-2 infection. SARS-CoV-2, severe acute respiratory syndrome coronavirus 2; PCR, polymerase chain reaction; RVP, respiratory viral panel; RSV, respiratory syncytial virus No differences were statistically significant

	SARS-CoV-2 Testing by PCR, No. (%)	
	Positive (n = 18)	Negative (n = 243)
RVP positive	4 (22.22)	91 (37.14)
Influenza		
A 2009, H1	0	1 (0.41)
B	0	2 (0.82)
RSV	1 (5.56)	10 (4.08)
Parainfluenza		
2	0	1 (0.41)
3	0	2 (0.82)
4	1 (5.56)	2 (0.82)
Adenovirus	0	19 (7.76)
Coronavirus		
HKU1	0	2 (0.82)
NL63	2 (11.11)	8 (3.27)
OC43	0	4 (1.63)
Human metapneumovirus	0	10 (4.08)
Human rhinovirus/Enterovirus	2 (11.11)	59 (24.08)
*Bordetella parapertussis*	0	1 (0.41)
*Chlamydia pneumoniae*	0	2 (0.82)
*Mycoplasma pneumoniae*	0	1 (0.41)

**Table 4:** Symptoms in children with co-infections versus those without co-infections, stratified by SARS-CoV-2 status by PCR. SARS-CoV-2, severe acute respiratory syndrome coronavirus 2; PCR, polymerase chain reaction Bold values indicate proportions of symptoms are significantly different (p < 0.001, Fisher’s exact test) among children with co-infections compared to without co-infections.

No. (%)	SARS-CoV-2 positive			SARS-CoV-2 negative	
	With co-infections	Without co-infections		With co-infections	Without co-infections
	N = 4	N = 14		N = 91	N = 154
Symptoms					
Fever	3 (75)	7 (50)		49 (54)	91 (59)
Cough	3 (75)	4 (29)		56 (62)	43 (28)
Shortness of breath	2 (50)	9 (64)		42 (46)	58 (38)
Nasal congestion/Rhinorrhea	2 (50)	3 (21)		36 (40)	20 (13)
Sore throat	0	1 (7)		6 (7)	4 (3)
Nausea/Vomiting	1 (25)	4 (29)		14 (15)	40 (26)
Diarrhea	0	0		4 (4)	12 (8)
Fatigue/Malaise/Weakness	1 (25)	3 (21)		2 (2)	4 (3)
Myalgia/Arthralgia	0	1 (7)		1 (1)	6 (4)
Rash/Skin lesions	0	0		5 (5)	12 (8)
Anosmia/Ageusia	0	2 (14)		0	0
Subjective fevers	0	1 (7))		3 (3)	6 (4)
Seizures	0	3 (21)		3 (3)	13 (8)
Other	0	1 (7)		4 (4)	7 (5)

**Table 5:** Comorbidities in children with versus without SARS-CoV-2 infection. SARS-CoV-2, severe acute respiratory syndrome coronavirus 2; PCR, polymerase chain reaction; HIE, hypoxic-ischemic encephalopathy ^a ^Fisher’s exact p-value reported.

	SARS-CoV-2 Testing by PCR, No. (%)			
	Positive (N = 18)	Negative (N = 245)	P-Value^a^	
Comorbidity	11 (61.11)	158 (64.49)	0.80	
Asthma/Reactive airway disease	3 (16.67)	21 (8.57)	0.22	
HIE	2 (11.11)	3 (1.22)	0.04	
Epilepsy	2 (11.11)	8 (3.27)	0.14	
Obesity	2 (11.11)	0	0.004	
Blood disease(s) or cancer(s)	2 (11.11)	44 (17.96)	0.75	
Hereditary spherocytosis	1 (5.56)	0	0.07	
Hemophilia A	1 (5.56)	0	0.07	
Solid tumor	0	11 (4.49)	0.99	
Sickle cell disease	0	11 (4.49)	0.99	
Leukemia	0	8 (3.27)	0.99	
Bone marrow transplant	0	5 (2.04)	0.99	
Immunosuppressant use	0	5 (2.04)	0.99	
Lymphoma	0	4 (1.63)	0.99	
Solid organ transplant	0	3 (1.22)	0.99	
Aplastic anemia	0	1 (0.41)	0.99	
Common variable immune deficiency	0	1 (0.41)	0.99	
Immune thrombocytopenia	0	1 (0.41)	0.99	
Lupus	0	1 (0.41)	0.99	
Prematurity	2 (11.11)	19 (7.76)	0.64	
Structural lung disease	1 (5.56)	21 (8.57)	0.99	
Diabetes	1 (5.56)	7 (2.86)	0.44	
Traumatic brain injury	1 (5.56)	2 (0.82)	0.19	
Febrile seizures	1 (5.56)	0	0.07	
Necrotizing enterocolitis	1 (5.56)	0	0.07	
Congenital heart disease	0	15 (6.12)	0.61	
Neuromuscular damage	0	11 (4.49)	0.99	
Failure to thrive	0	4 (1.63)	0.99	
Cystic fibrosis	0	3 (1.22)	0.99	
Cerebrovascular disease	0	2 (0.82)	0.99	
Arnold-Chiari malformation	0	2 (0.82)	0.99	
Sickle cell trait	0	1 (0.41)	0.99	
End stage renal disease	0	1 (0.41)	0.99	
Short gut syndrome	0	1 (0.41)	0.99	
Spina bifida	0	1 (0.41)	0.99	
Vesicoureteral reflux	0	1 (0.41)	0.99	
